# Jumping on the Edge—First Evidence for a 2 × 6-meric Hemocyanin in Springtails

**DOI:** 10.3390/biom9090396

**Published:** 2019-08-22

**Authors:** Juliane Schmidt, Heinz Decker, Michael T. Marx

**Affiliations:** 1 Institute for Molecular Physiology, Johannes Gutenberg-University Mainz (JGU), 55128 Mainz, Germany; 2 Institute for Zoology, Johannes Gutenberg-University Mainz (JGU), 55128 Mainz, Germany

**Keywords:** hemocyanin, springtails, Collembola, Crustacea, terrestrialization, hexapods

## Abstract

Hemocyanins are respiratory dioxygen carrier proteins found in many arthropods including ancient terrestrial species such as spiders and scorpions as well as marine horseshoe crabs. As hemocyanins are highly conserved in this lineage, it is possible to observe an evolutionary descent through its subunits and their overall structure. Unfortunately, little is known about the structure and function of hexapod hemocyanins. Using recent springtail taxa (Collembola) as models for basal hexapods, and the help of electron microscopy, light scattering, SDS PAGE, and Western blot, we could demonstrate for the first time the presence of 2 × 6-meric hemocyanins in the hemolymph of hexapods. The quaternary structure is composed of at least two different subunits and looks nearly identical to the hemocyanin found in decapod crustaceans. In addition, homology modeling and western blotting suggest a close structural relationship between collembolan and crustacean hemocyanin. Such a respiratory protein was possibly helpful in the early terrestrialization process of ancient Collembola. In addition, physiological adaptations to hypoxic or temporarily anoxic conditions could be a possible explanation for the presence of this respiratory protein. Nevertheless, it has to be concluded that the primary benefit of hemocyanin for springtails remains unclear.

## 1. Introduction

Hexapoda comprise of the well-known class Insecta with a second one called Entognatha. Entognatha contain three of the five formerly apterygote taxa: Springtails (Collembola), two-pronged bristletails (Diplura), and coneheads (Protura). It is unknown when ancient Collembola first occurred but the fossil springtail *Rhyniella praecursor* [1] is considered to be one of the oldest terrestrial arthropod taxa from about 420 to 400 Myr ago [2,3,4]. Collembola inhabit every soil layer and the soil surface in very high abundances, making them a key group among the soil arthropods. Over their long evolutionary history, they were able to colonize different specialized and sometimes extreme habitats (e.g., water surfaces, sand coasts, deserts, the Arctic, and the Antarctic) [5,6,7,8,9,10,11,12], which makes them one of the most ecologically, diversified, and distributed arthropod groups [13].

Hemocyanins (hc) are highly conserved respiratory proteins in the lineage of arthropods and molluscs. They occur freely dissolved in the hemolymph (hl) and especially for arthropods, they consist of subunits of ca. 72–74 kDa which connect themselves to hexamers. Depending on the species, they can be found as single hexamers or as multiples of hexamers [14,15]. Looking at the quarternary structure of arthropod hemocyanins, they are always composed of a multiple of hexamers. Horseshoe crabs, spiders, and crustaceans usually form 8 × 6-mers, 4 × 6-mers, and 2 × 6-mers, respectively [15]. Insects or hexapods are thought to possess a single hexamer, which, although functional, is very poorly distinguished from non-respiratory hexamerin [16]. Recent investigations also focused on the immunological properties of hemocyanin in arthropods. The antibacterial activity of malacostracean hemocyanin against clinical pathogens or molecular diversity changes of the white shrimp hemocyanin related to pathogen infection are current examples [17,18].

Recently, hemocyanin was identified in Collembola hemolymph by RT-PCR and RACE techniques, leading to a full-length sequence for *Sinella curviseta* hemocyanin (termed ScuHc1) and a partial sequence from *Folsomia candida* hemocyanin (FcaHc1) [19]. Both were classified as hexapod hemocyanin subunit type 1 based on about 60% sequence identity with the subunit type 1 hemocyanin from the stonefly *Perla marginata* [20], which was shown to be a biologically active 1 × 6-meric hemocyanin during the stonefly’s larval stadium [20].

It has not been possible, so far, to present the structure of a hexapod hemocyanin. With this work on a basal and very old hexapod clade, we want to suggest the collembolan hemocyanin quaternary structure. Some ideas concerning the function of this respiratory protein in Collembola will be discussed.

## 2. Materials and Methods

### 2.1. Animals and Hemolymph Collection

In order to gain structural information about springtail hemocyanin, we searched for it in the hemolymph of the tiny springtail species *Sinella curviseta*, *Folsomia candida*, *Coecobrya tenebricosa*, *Isotoma viridis*, *Orchesella cincta*, and *Orchesella villosa*. The hemolymph samples were freshly collected before performing the experiments. The animals were anesthetized in the freezer at −24 °C for 5 min. The protein samples were immediately obtained by decapitation of the anesthetized animals with a scalpel and immersion of the two body parts in a 15 µL drop of a 0.1 M Tris/HCl buffer (pH 7.8, 10 mM CaCl_2,_ and MgCl_2_ each) in the presence of protease inhibitors (Protease Inhibitor Cocktail, Sigma) for 20 s. The same 15 µL drop was used for the next 15 individuals as well. This process was repeated with new individuals and new drops until the amount of protein was sufficient for the planned experiment. Only the electron microscopic images were be performed with all listed Collembola species. Since the sequence of ScuHc1 is known, we focused on the hemocyanin from *Sinella curviseta* for the other experiments. 

### 2.2. Protein Determination

Due to the impurity of the samples, the protein concentration could only be roughly estimated. A UV/Vis spectrum was recorded on a NanoDrop ND-1000 photo spectrometer (PEQLAB Biotechnologie, Erlangen, Germany). The concentration was determined under the assumption that an impure protein solution of 1 mg/mL has an absorption of 1 at 280 nm. For a 15 µL protein drop obtained from 15 individuals as described above, this resulted in an approximate concentration of 0.1 mg/mL. Due to the very low protein yield and the small sample volume, a more accurate calculation of the protein concentration was not performed.

### 2.3. Electron Microscopy

For the negative staining electron microscopy, the hemolymph of 15 individuals of each species was used and processed as previously described [21]. Larger particles were removed from the samples by centrifugation for 20 min and at 22,000 g. The hemolymph was applied to glowing continuous carbon carrier layers and stained with aqueous 2% (w/v) uranyl format using the single-drop method [22]. A Technai12 electron microscope and a connected MegaView III camera were used for the electron microscopic images. Using 13 different 2 × 6-meric objects with exactly the same position and orientation on the EM images, the software EM-Menu (TVIPS, Gauting, Germany) was used to create a 2D average image. This class sum was one of 184, which were calculated out of 1258 picked 2 × 6-meric objects on 100 images. The class sum of this orientation was chosen because it best reflects the typical structure of 2 × 6-meric hemocyanin.

### 2.4. SDS-PAGE and Immunoblotting

Denaturing SDS-PAGE was carried out as 7.5% gels under standard conditions [23]. For each sample, the hemolymph of 15 individuals from *Sinella curviseta* and 15 µL buffer were used and denaturing buffer was added 1:1. The gels were stained with Coomassie dyeing after Kang [24].

For western blotting, the proteins were transferred onto nitrocellulose membranes. Non-specific binding sites were blocked for 45 min using 4% non-fat dry milk in TBST (10 mM Tris/HCl, pH 7.5, 140 mM NaCl, 0.3% Tween 20). Primary anti-hemocyanin or anti-hemolymph antibody serum, diluted 1:10,000 in blocking solution was carried out overnight. The membrane was then washed four times in a row with TBST. Afterwards, two hours of incubation with secondary antibodies (goat-anti-rabbit IgGs coupled with alkaline phosphatase, diluted 2:20,000 with TBST) was performed. The membrane was finally washed four times with TBST, before staining with NBT and BCIP. All steps, except the detection, were performed at a temperature of 4 °C.

### 2.5. Multi-Angle Laser Light Scattering

The hemolymph of 300 individuals of *Sinella curviseta* in 100 µL buffer was used for this method because it requires a very high protein concentration. With this concentration, we just reached a credible detection range to calculate the molecular weight of *ScuHc*. After the purification by size exclusion gel chromatography (Superose 6 10/300 GL SEC, GE Healthcare, Chicago, IL, USA), the Multi-Angle Laser Light scattering (MALLS, Wyatt Technology, Santa Barbara, CA, USA) of the proteins was performed. BSA monomer (Sigma-Aldrich, St. Louis, MO, USA) was used to calibrate the MALLS instrument by normalization of the different detector signals with respect to the 90° detector signal. The flow rate was 0.4 mL/min and the molecular weight was calculated using the Exzess-Rayleigh ratio by the software ASTRA (Wyatt Technology, Santa Barbara, CA, USA). A correction factor containing the molecular weight and the extinction coefficient of the subunits was used for calculation. Both were determined with Protparam from the existing *Sinella curviseta* hemocyanin primary sequence [19,25].

### 2.6. Mass Spectrometry Sample Preparation

The protein identification was performed by the IMB proteomics core facility (Mainz, Germany). Protein lanes were cut out of the SDS-PAGE gel, crushed, destained in 50% ethanol in 25 mM ammonium hydrogen carbonate (room temperature, rotated for 15 min), and dehydrated in 100% acetonitrile (room temperature, rotated for 10 min). Dehydrated samples were trypsin-digested (1 µg trypsin/sample in 50 mM Triethylammonium bicarbonate buffer pH 8.0) at 37 °C for 12 h. Stepwise peptide extraction was done as follows: Twice extraction solution (30% acetonitrile) and 100% acetonitrile centrifuged for 15 min (25 °C, 1400 rpm). After purification and desalting using C18 StageTips (Empore, Sigma-Aldrich, St. Louis, MO, USA) [26], 3.5 µL peptides were loaded and separated on C18 column (75 µm inner diameter, New Objective) The column was self-packed with 1.9 µm Reprosil beads (Dr. Maisch) and mounted to an Easy LC 1000 HPLC (Thermo Fisher Scientific, Waltham, MA, USA). 

### 2.7. Mass Spectrometry Measurement and Data Analysis

Reverse phase chromatography was performed with 0.1% formic acid (buffer A), 80% acetonitrile, and 0.1% formic acid (buffer B). Peptides were eluted under the following gradient: 0–21 min 0–22% buffer B, 21–28 min 22–40% buffer B, 28–32 min 40–95% buffer B; and directly sprayed into a Q Exactive Plus mass spectrometer (Thermo Fisher Scientific, Waltham, MA, USA). The mass spectrometer was operating in positive scan mode with a full scan resolution of 70,000; AGC target 3 × 10^6^; max IT = 20 ms; scan range 300–1650 *m/z*; and a Top10 MSMS method. Normalized collision energy was set to 25 and MSMS scan mode operated with resolution of 17,000; AGC target 1 × 10^5^; max IT = 120 ms.

A database search was performed using MaxQuant Version 1.5.2.8 [27] against Uniprot Collembola, *Manduca secxta*, *Carausius morosus*, and insecticyanin database (downloaded 15th October 2015, 2315 entries, FASTAfile attached in the supplementary) with Trypsin/P as a digestion enzyme (2 missed cleavages allowed). The following settings were used: The variable modifications were; Acetyl (Protein N-term) and Oxidation (M), and the fixed modifications were; Carbamidomethyl (C), FDR of 1% on peptide, and protein level.

Proteins were considered to be identified if they matched at least two peptides (one of them unique). 

Proteins that correspond to the reverse database or the common contamination list and proteins with peptides identified only by modified peptides were filtered out. Further bioinformatic analyses were performed in R [28] using the following libraries: Knitr, plyr, reshape, ggplot2, and psych.

## 3. Results and Discussion

**Figure 1:**
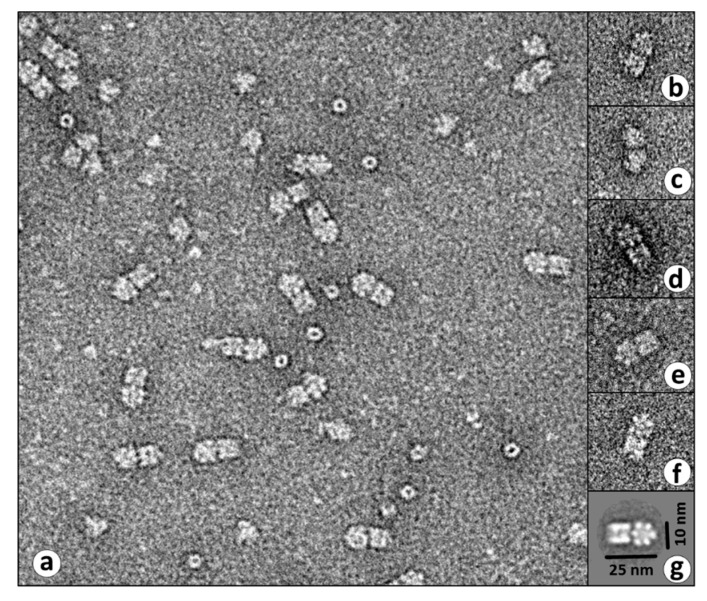
Electron microscopical images of hemolymph proteins of various springtails. The “single droplet negative staining“ technique and 2% (v/v) aqueous uranyl formate was applied for the preparation of the sample. (**a**) Overview of the hemolymph proteins of *Orchesella villosa*. A huge amount of the 2 × 6-meric protein is present in the hemolymph. Inserts show 2 × 6-meric proteins found in the hemolymph from *Isotoma viridis* (**b**), *Folsomia candida* (**c**), *Coecobrya tenebricosa* (**d**), *Orchesella cincta* (**e**), and *Sinella curviseta* (**f**) [29]. (**g**) Superposition of 13 images of *Sinella curviseta* hemocyanin for gaining a better contrast.

Analysis of the hemolymph by transmission electron microscopy demonstrated hemocyanin-like structures in every species (Figure 1). They were characterized as 2 × 6-mer and strongly reminiscent to the hemocyanins found in *Astacus leptodactylus* (crayfish), *Homarus americanus* (lobster), and *Cancer paragurus* (crab) [29]. Here, two hexamers dimerize, being rotated by 90° against each other (Figure 1g). Since the sequence of ScuHc1 is known, we focused on the hemocyanin-like protein, from *Sinella curviseta*. Multi-Angle Laser Light scattering of the proteins purified by size exclusion gel chromatography revealed a molecular mass of app. 850 kDa (Supplementary Figure S1) which is in the order of 2 × 6-meric hemocyanins being built up by subunits with typical masses ranging between 73–83 kDa [30]. Smaller particles were also found which could not be assigned to 1 × 6-meric hemocyanins due to their form and size.

**Figure 2:**
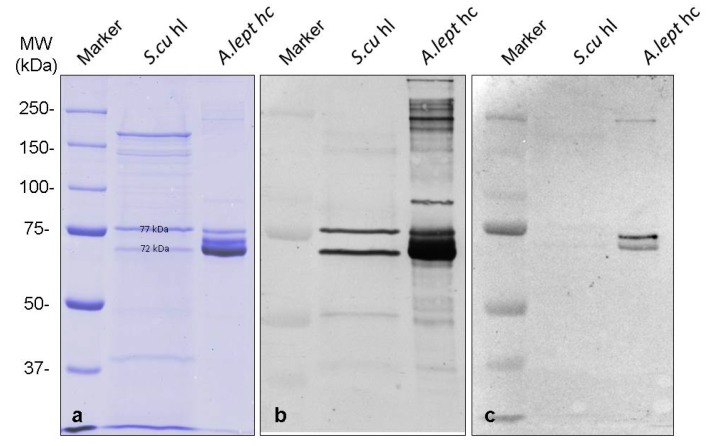
Identification of two hemocyanin subunit types in *S. curviseta* hemolymph by Western blotting. (**a**) SDS-PAGE (pH: 8.8, 7.5%). Marker: Biorad Precision Plus Unstained Standards. S.cu hl = *Sinella curviseta* hemolymph. A. lep hc = *Astacus leptodactylus* hemocyanin. (**b**) Western blot with antibodies against hemolymph proteins from crab *Astacus leptodactylus*. Two bands at about 72 and 77 kDa from *Sinella curviseta* hemolymph are strongly recognized, while various proteins from semi-purified *A. leptodactylus* hemocyanin are observed. (**c**) Western blot with antibodies against purified tarantula hemocyanin. Two strong bands of semi-purified *A. leptodactylus* hemocyanin can be observed but only two very weak bands from *Sinella curviseta* hemolymph bands can be detected. Marker: Biorad Precision Plus Dual Color Standards. Blot was scanned black and white.

Two different bands from the SDS-PAGE (Figure 2a) with about 72 and 77 kDa were investigated by LC-MS/MS to identify the proteins. Unique peptides (six of the 77 kDa and two of the 72 kDa bands) match the ScuHc1 sequence (Figures S2 and S3) [19]. Thus, the 2 × 6-meric protein molecules of *Sinella curviseta* are hemocyanins, built by two different hemocyanin subunit types with 72 kDa and 77 kDa. The latter one correlates the known ScuHc1, while we annotated the other one as ScuHc2, which has not been detected in springtails before. The question arises whether and how its sequence differs from the previously known subunit type 2 [19] and whether the ScuHc2 subunit is responsible for the formation of 2 × 6-mers, as it has already been shown for some crustaceans hemocyanins [15,29]. Also of great interest would be how this missing sequence is placed in the phylogenetic studies in order to further elucidate the evolution of hemocyanins. Further investigations are necessary to determine the complete sequence of subunit ScuHc2 and to answer this question. For example, the complete amino acid sequence and the glycosylation of the hemocyanins from the malacostracean taxa *Carcinus aestuarii* and *Eriphia verrucosa* helped to understand the quaternary structure of the molecules [31,32]. These analyses could also help to gain a better understanding of the quaternary structure of hexapod hemocyanins. 

To obtain information about the immunological relationship, antibodies against the 2 × 6-mer hemocyanins of crayfish *Astacus leptodactylus* and the 4 × 6-meric hemocyanins of tarantula *Eurypelma californicum* were used. These references were chosen because both animals are well studied model organisms in hemocyanin research [14,15,29]. Antibodies raised against hemolymph (hl) proteins from *A. leptodactylus* strongly recognize *Sinella curviseta* hemocyanins (Figure 2b), while almost no cross-reactivity was observed with antibodies raised against purified 4 × 6-meric *E. californicum* hemocyanins (Figure 2c). Thus, Western blots reveal a close immunological relationship of the two subunit types from *Sinella curviseta* with crustacean hemocyanins. The bands of S.cu hl always showed a significantly broader expression compared to other samples on the SDS-PAGE. This is probably due to the fact that these samples had to be applied unpurified and therefore contained tissue fluid, small cell debris, and other macromolecules in addition to the different proteins of the hemolymph.

To summarize, negative staining electron microscopic images, light scattering, Western plots, and the protein identification by LC-MS/MS prove that the observed proteins are 2 × 6-meric hemocyanins, composed of two different subunits, occur in the hemolymph of the springtail *Sinella curviseta*. Furthermore, the EM images of the hemolymph of other springtail species (Figure 1) imply that the 2 × 6-meric hemocyanin is dominant throughout different families of Collembola.

Hemocyanin is the main respiratory protein in Pancrustacea and it is discussed that the ancestors of terrestrial hexapods used hemocyanin in addition to their gills and tegumentary respiration to establish their life outside of water [30]. We suggest that collembolan hemocyanin has a cooperative oxygen affinity due to its 2 × 6-meric structure as it has been shown for their malacrustacean equivalents [15], and that springtails retained the 2 × 6-meric hemocyanin of their crustacean ancestor possibly as a plesiomorph feature, which was helpful in the early terrestrialization phase of this taxon. The function of hemocyanin as a respiratory protein could be linked to physiological adaptations in Collembola species inhabiting deeper layers of the soil, sand coasts, or tidal zones. Different collembolan species showed a metabolic shift under anaerobic conditions (anoxia) [33]. *Folsomia candida* has distinctly elevated lactate levels following artificially induced anoxia [34,35,36]. Additionally, an increased heart rate was measured in this species during hypoxic conditions. This blood circulation modulation helps to maintain the partial pressure between the medium, the blood, and the tissue [37]. Both adaptations allow individuals of *F. candida* a fully functional respiration performance even during extreme low partial pressures of oxygen (down to 6.666 Pa) [36]. In *Anurida maritima* (Guérin-Méneville, 1836) (Neanuridae, Collembola), the partial pressure of oxygen under extreme conditions can be even lower [38]. This species prefers habitats in tidal zones and should be well adapted to periodic flooding events. At low tides, these animals feed on fine substrates such as the algae and suspended sediments of the surface. With increasing water levels, individuals gather in aggregations (“nests”) under stones in order to survive the flooded period in a commonly used air pocket [39]. Exposed to such changing environmental conditions, the possibility to use very low partial pressures of oxygen (as low as 1000 Pa) makes this species highly competitive. A critical oxygen level for these animals was not achievable during experiments performed by Zinkler et al. [38]. However, at 1000 Pa, a small reduction of the oxygen uptake could be measured, but without causing serious problems for the animals concerning the regulation of the oxygen uptake. These features coupled with an effective respiratory protein as an oxygen reservoir could increase the survival abilities of these species in partially hypoxic or even anoxic conditions as can occur in deeper soil layers or tidal zones.

The ability to survive longer periods of flooding in the egg stage was documented for *Isotomiella minor* (Schäffer, 1896) (Isotomidae, Collembola) and several members of the taxon Symphypleona (Collembola) [40,41]. Some of these species have the ability to survive embryonic and post-embryonic development as well as the first molt submerged (semiaquatic lifestyles) [42]. All these adaptations to low oxygen environments could also imply the need for a respiratory protein like hemocyanin. 

Nevertheless, despite these assumptions it remains unclear why hemocyanins are present in Collembola. They do not seem to be needed as respiratory proteins in the majority of species, as the supply with oxygen by diffusion (known as tegumentary respiration [15]) is sufficient to meet the respiratory demands. This is also supported by the lack of trachea in *Sinella curviseta* [43]. However, the mRNA sequence of ScuHc1 [19] contains all six histidines coordinating the two copper atoms at the active site of type 3 copper proteins. As we could not detect the typical absorbance peak at 340 nm for bound dioxygen (Supplementary Figure S4), it seems likely that Collembola hemocyanin occurs in the met state, as often observed for hemocyanins [44]. Further experiments could address this question if it is possible to gain enough intact hemolymph from these extremely small animals.

In summary, this study showed the occurrence of a 2 × 6-meric quaternary hemocyanin structure consisting of at least two different subunits in all investigated species of the taxon Collembola. The benefit of hemocyanin for springtails remains unclear, but the early terrestrialization process or physiological adaptation to hypoxic or transient anoxic conditions may be possible explanations for the presence of this respiratory protein.

## Supplementary Materials

The following are available online at https://www.mdpi.com/2218-273X/9/9/396/s1, Figure S1: Absorbance of size exclusion gel chromatography and diffraction of the Multi Angle Laser Light scattering (MALLS) and SDS-PAGE of the collected Peaks, Figure S2: Protein identification of the 72 and 77 kDa bands out of the SDS-PAGE of Sinella curviseta hemolymph (Figure 2) by mass spectroscopy, Figure S3: MSMS spectrum masses of the Protein identification of *S.cu* hemocyanin by mass spectroscopy, Figure S4: UV/Vis absorption spectrum of *Sinella curviseta* hemolymph. Data S6: Detailed data of the performed protein identification as compressed data incl.FASTA file of the databases which were used for the protein identification by mass spectroscopy. Uniprot Collembola, *Manduca secxta*, *Carausius morosus* and insecticyanin database (downloaded 15th October 2015, 2315 entries.
