# Supplementary material for: Jumping on the Edge—First Evidence for a 2 × 6-meric Hemocyanin in Springtails
Source: Biomolecules. 2019 Aug 22;9(9):396. doi: 10.3390/biom9090396 (PMC6769593; doi:10.3390/biom9090396)
Supplement: Supplementary file 1 [file biomolecules-09-00396-s001.zip › biomolecules-551611-supplementary.pdf]

biomolecules

Supplementary materials

Jumping on the Edge—First Evidence for a 2 × 6-meric Hemocyanin in Springtails

Juliane Schmidt [1,*], Heinz Decker [1] and Michael T. Marx [2]

[1] Institute for Molecular Physiology, Johannes Gutenberg-University Mainz (JGU), 55128 Mainz, Germany
[2] Institute for Zoology, Johannes Gutenberg-University Mainz (JGU), 55128 Mainz, Germany
* Correspondence: julschm@uni-mainz.de

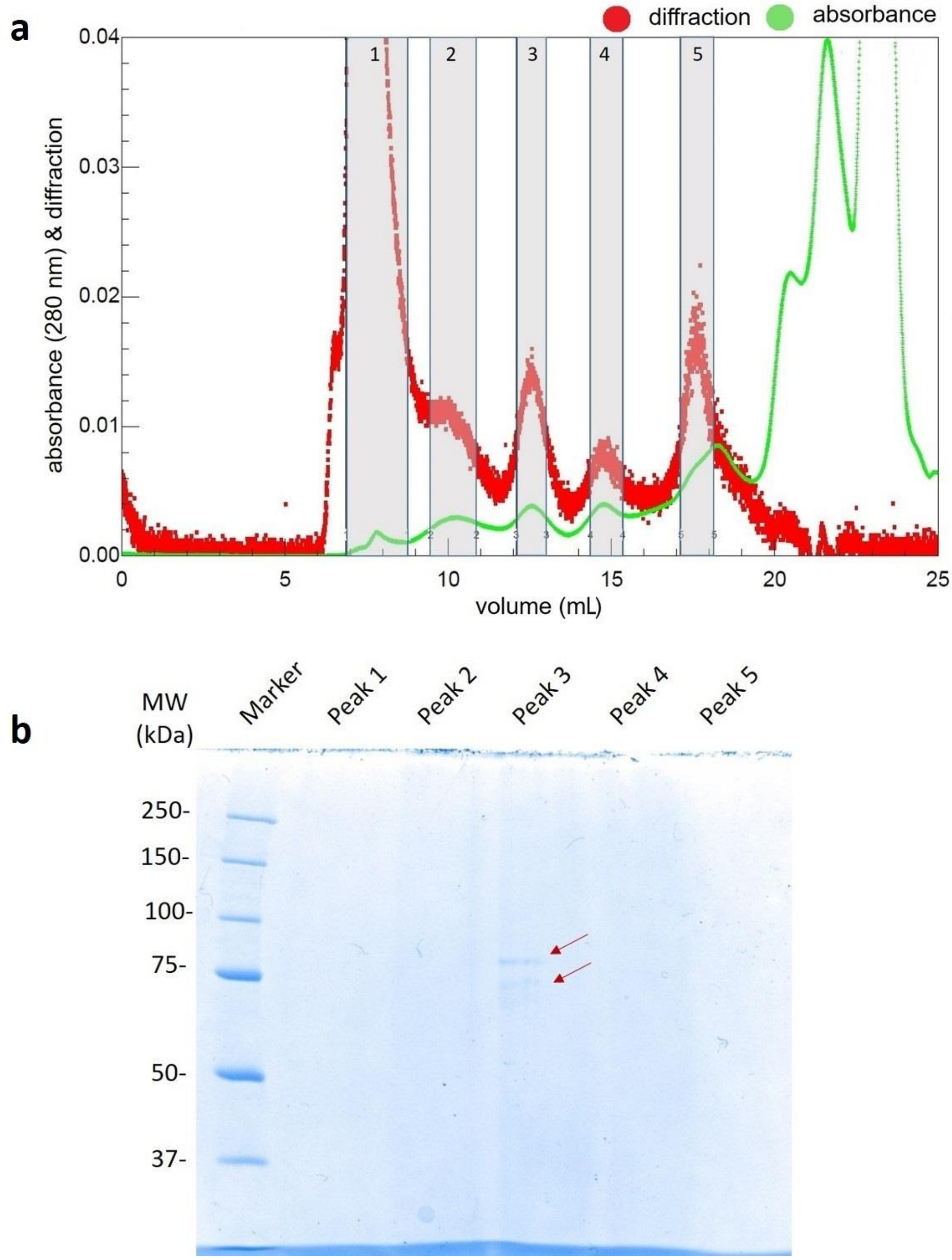

Figure S1. Absorbance of size exclusion gel chromatography and diffraction of the Multi-Angle Laser Light scattering (MALLS) and SDS-PAGE of the collected Peaks (**a**). The hemolymph of 300 individuals of *Sinella curviseta* in 100 µL buffer was used for this method which reached a credible detection range to calculate the molecular weight of *ScuHc*. MALLS (Wyatt Technology, USA) of the proteins was performed after the purification by size exclusion gel chromatography (Superose 6 10/300 GL SEC, GE Healthcare). Flow rate = 0.4 mL/min. (**b**) 7.5% SDS-PAGE, pH: 8.8. Marker: Biorad Precision Plus Dual Color Standards. Coomassie dye after Kang [1]. The concentrated peaks 1-5 from the MALLS experiment were applied. Only Peak 3 shows bands on the gel. They correspond to the typical size of hemocyanin subunits (~75 kDa). The other traces showed no bands, which is probably due to a too low protein concentration or the possibility that the peaks did not contained proteins at all. Since this question was not beneficial to the actual project, this was not pursued further. The molecular weight of Peak 3 was calculated using the Exzess-Rayleigh ratio by the software ASTRA (Wyatt Technology, USA) and was app. 850 kDa. A correction factor containing the molecular weight and the extinction coefficient of the subunits was used for calculation. Both were determined with Protparam from the existing *Sinella curviseta* hemocyanin primary sequence [2,3].

```
                   1                                                                              80
ScuHc1 (B9W4M7)   MARFALTLIACTLVVAVAATPADQEFLTKQKEIVKLLNKVHELNFYQDQATIGKDWDPLAHLDSYKNVRVVKELVKELKN
   77 kDa band   --------------------------------------------------------------------------------
   72 kDa band   --------------------------------------------------------------------------------

                  81                                                                             160
ScuHc1 (B9W4M7)   GKLIKRGEIFNLFNEEHRREMILLFETLFFAKDWDTFHKTACWARDKINEGQFVYALSVATLHRADTRGIRLPPAYETYP
   77 kDa band   -------------------EMILLFETLFFAKDWDTFHK------DKINEGQFVYALSVATLHR-------LPPAYETYP
   72 kDa band   -------------------EMILLFETLFFAK-------------DKINEGQFVYALSVATLHR-------LPPAYETYP

                 161                                                                             240
ScuHc1 (B9W4M7)   HLFVTSQVIHEAYAAKMRQEPAVIHMNFTGTVRNPEQRVAYFGEDVGMNSHHAVFHMDWPFWWNEEKYGLTKDRKGELFW
   77 kDa band   HLFVTSQVIHEAYAAK----------------------------------------------------------------
   72 kDa band   HLFVTSQVIHEAYAAK----------------------------------------------------------KGELFW

                 241                                                                             320
ScuHc1 (B9W4M7)   YMHHQLITRFDAERLSNDLNEVEALHWDKPIVEGFYPQTTYRKGGEFPARPDNFMFHDLKDHRVADLEAYEERIREAISA
   77 kDa band   --------------------------------------------------------------------------------
   72 kDa band   YMHHQLITR-----------------------------------------------------------------------

                 321                                                                             400
ScuHc1 (B9W4M7)   GVIFDAHDPNTFISLNNTEGIDKLGAIIEASSCSINPSFYGSLHNLGHIILGRVVDPLGKFGMPPGVMEHFETATRDPAF
   77 kDa band   ------------------------------------------------------------FGMPPGVMEHFETATR----
   72 kDa band   ------------------------------------------------------------FGMPPGVMEHFETATR----

                 401                                                                             480
ScuHc1 (B9W4M7)   FRLHKHIDNIFKEHKDSLVPYTHEELDVEGVDIKNVEVDDLVTYFEDFDIDMLNALDDAAGLTDVDIKARVQRLNHKPFG
   77 kDa band   -------------------------------------------------------------------------LNHKPFG
   72 kDa band   --------------------------------------------------------------------------------

                 481                                                                             560
ScuHc1 (B9W4M7)   IKIIANSAAEKTVTVRLFLAPKYDWYGREVPLDIQRWKFIELDKFAVKLTAGENAIVRKSSESSVTIPDPVSTHDLRKLV
   77 kDa band   IK------------------------------------------------------------------------------
   72 kDa band   --------------------------------------------------------------------------------

                 561                                                                             640
ScuHc1 (B9W4M7)   DDAIAGTATLEVDKDVRHCGVPDRLLLPKGKTNGMKYTMFVMLSDFEEDKVNDLPHDYEYGGFVSYCGTINHKYPDKKPM
   77 kDa band   -----------------------------------------------------------------------------KPM
   72 kDa band   --------------------------------------------------------------------------------

                 641                             672
ScuHc1 (B9W4M7)   GWPLDRKICKDSFHDVTNMYFRDVEIKFEHTH
   77 kDa band   GWPLDR--------------------------
   72 kDa band   --------------------------------

    List of peptides 77 kDa band:            List of peptides 72 kDa band:
     start   end sequence                     start   end sequence
       100   112 EMILLFETLFFAK                    100   112 EMILLFETLFFAK
       113   119 DWDTFHK                          126   144 DKINEGQFVYALSVATLHR
       126   144 DKINEGQFVYALSVATLHR              152   176 LPPAYETYPHLFVTSQVIHEAYAAK
       128   144 INEGQFVYALSVATLHR                235   249 KGELFWYMHHQLITR
       152   176 LPPAYETYPHLFVTSQVIHEAYAAK        381   396 FGMPPGVMEHFETATR
       381   396 FGMPPGVMEHFETATR
       474   482 LNHKPFGIK
       638   646 KPMGWPLDR
```

Figure S2. Protein identification of the 72 and 77 kDa bands out of the SDS-PAGE of *Sinella curviseta* hemolymph (Figure 2) by mass spectroscopy (MS). To unequivocally confirm that these two bands are hemocyanin, we analyzed these peptides by MS protein identification and clearly matched both proteins to the ScuHc sequence [3] by mapping eight peptides of the 77 kDa band (including six unique ones) and five peptides of the 72 kDa band (including two unique ones). The various sequences of the peptides are given with the position in the ScuH1 sequence. The protein identification was performed by the IMB proteomics core facility (Mainz, Germany) with a Thermo Q Exactive Plus mass spectrometer coupled to an EASY LC 1000 HPLC system. Data analysis software MaxQuant was used.

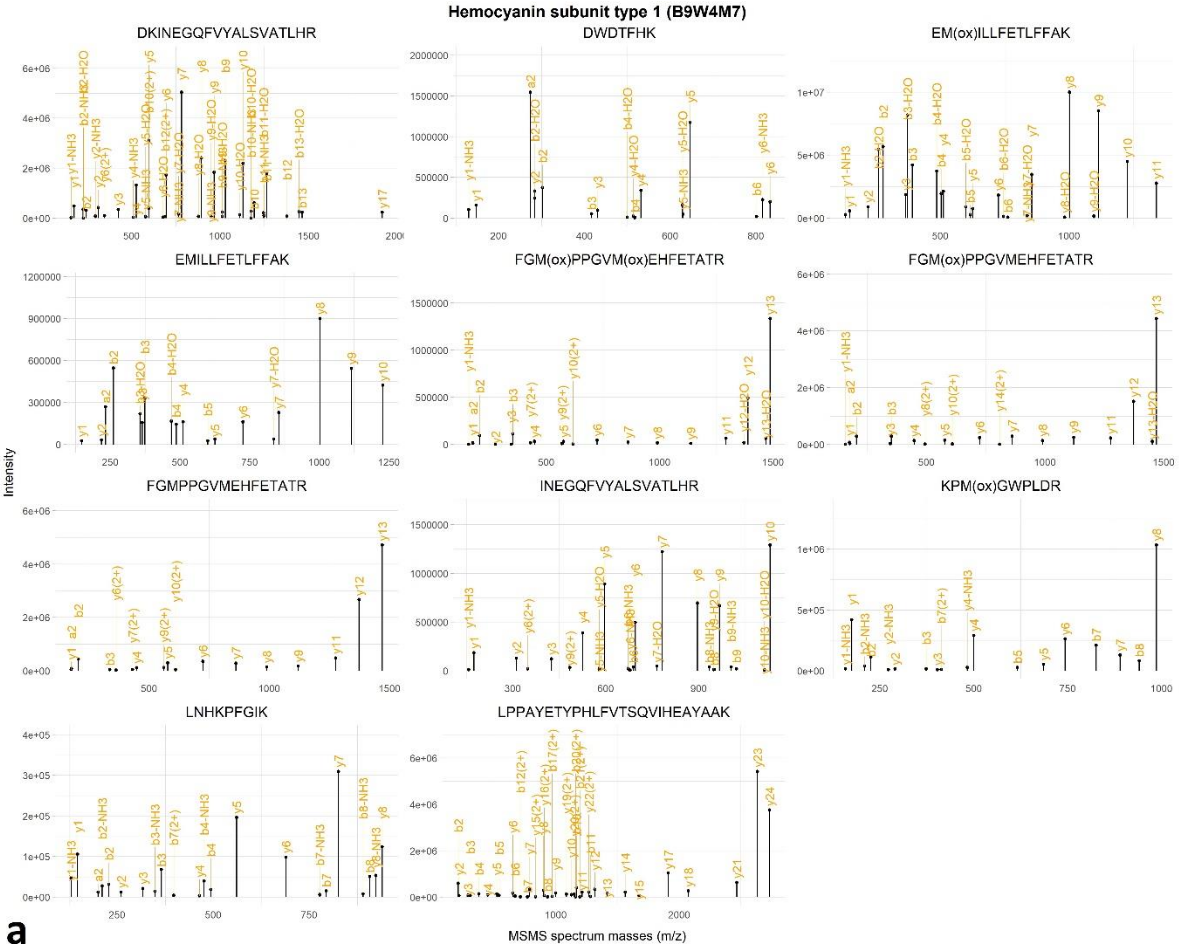

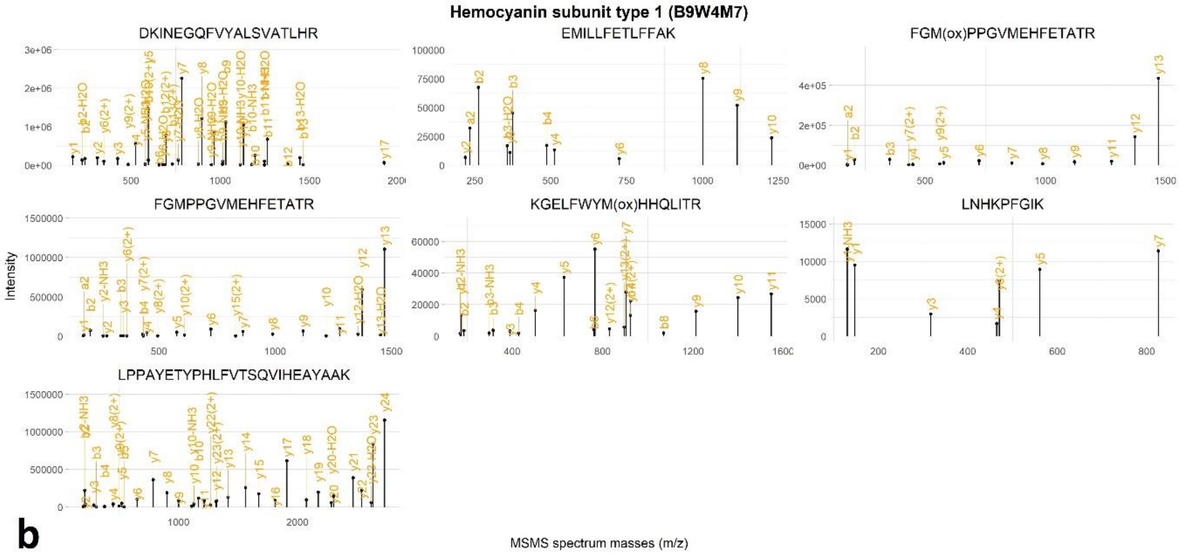

Figure S3. MSMS spectrum masses of the Protein identification of *S.cu* hemocyanin by mass spectroscopy. To confirm that the 72 and 77 kDa bands out of the SDS-PAGE (Figure 2) are hc, we analyzed these peptides by MS protein identification and matched both proteins to the ScuHc sequence (UniProt accession: B9W4M7) [2]. (**a**) MSMS spectrum masses (*m*/*z*) of the 77 kDa band. (**b**) MSMS spectrum masses (*m*/*z*) of the 72 kDa band. The protein identification was performed by the IMB proteomics core facility (Mainz, Germany) with a Thermo Q Exactive Plus mass spectrometer coupled to an EASY LC 1000 HPLC system. Data analysis software MaxQuant was used.

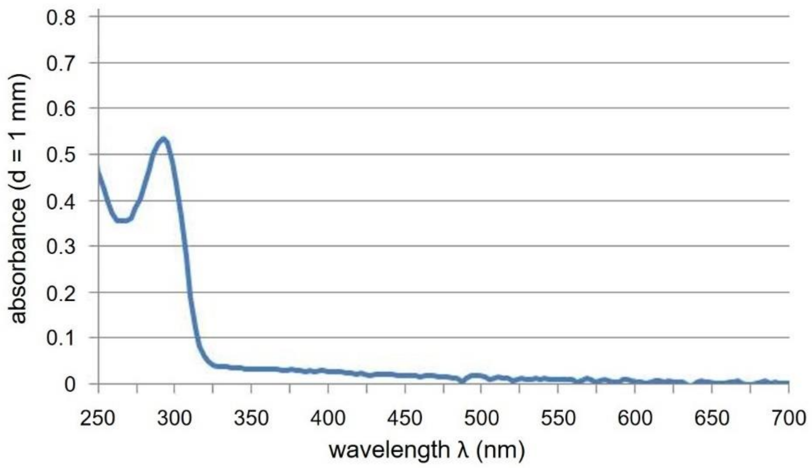

Figure S4. UV/Vis absorption spectrum of *Sinella curviseta* hemolymph recorded on a NanoDrop ND-1000 photo spectrometer (PEQLAB). The path length was 1 mm. Unpurified extract from 50 individuals in 15 µL buffer (0.1 M Tris/HCl, pH 7.8, 10 mM $CaCl_2$ and $MgCl_2$ each) was used. The sample was obtained as described in the main text. The characteristic 340 nm peak, which represents the oxygenated copper center of the hemocyanins, is not present. This peak could not be detected for any of the springtail species investigated.

References

1. Kang, D.; Gho, Y.S.; Suh, M.; Kang, C. Highly Sensitive and Fast Protein Detection with Coomassie Brilliant Blue in Sodium Dodecyl Sulfate-Polyacrylamide Gel Electrophoresis. *Bull. Korean Chem. Soc.* **2002**, *23*, 1511–1512, doi:10.5012/bkcs.2002.23.11.1511.
2. Pick, C.; Schneuer, M.; Burmester, T. The occurrence of hemocyanin in Hexapoda. *FEBS J.* **2009**, *276*, 1930–1941, doi:10.1111/j.1742-4658.2009.06918.x.
3. Gasteiger, E.; Hoogland, C.; Gattiker, A.; Duvaud, S.; Wilkins, M.R.; Appel, R.D.; Bairoch, A. Protein Identification and Analysis Tools on the ExPASy Server. In *The Proteomics Protocols Handbook*; Walker, J.M., Ed.; Humana Press: Totowa, NJ, USA, 2005; pp. 571–607, doi:10.1385/1-59259-890-0:571.

© 2019 by the authors. Licensee MDPI, Basel, Switzerland. This article is an open access article distributed under the terms and conditions of the Creative Commons Attribution (CC BY) license (http://creativecommons.org/licenses/by/4.0/).
